# An Anterior Cingulate Cortex-to-Midbrain Projection Controls Chronic Itch in Mice

**DOI:** 10.1007/s12264-022-00996-6

**Published:** 2022-12-17

**Authors:** Ting-Ting Zhang, Su-Shan Guo, Hui-Ying Wang, Qi Jing, Xin Yi, Zi-Han Hu, Xin-Ren Yu, Tian-Le Xu, Ming-Gang Liu, Xuan Zhao

**Affiliations:** 1 Department of Anesthesiology, Shanghai Tenth People’s Hospital, School of Medicine, Tongji University, Shanghai, 200072 China; 2 Department of Anatomy and Physiology, Shanghai Jiao Tong University School of Medicine, Shanghai, 200025 China; 3 Center for Brain Science of Shanghai Children’s Medical Center, Shanghai Jiao Tong University School of Medicine, Shanghai, 200127 China

**Keywords:** Anterior cingulate cortex, Chronic itch, Ventral tegmental area, Chemogenetics, Neural circuit

## Abstract

Itch is an unpleasant sensation that provokes the desire to scratch. While acute itch serves as a protective system to warn the body of external irritating agents, chronic itch is a debilitating but poorly-treated clinical disease leading to repetitive scratching and skin lesions. However, the neural mechanisms underlying the pathophysiology of chronic itch remain mysterious. Here, we identified a cell type-dependent role of the anterior cingulate cortex (ACC) in controlling chronic itch-related excessive scratching behaviors in mice. Moreover, we delineated a neural circuit originating from excitatory neurons of the ACC to the ventral tegmental area (VTA) that was critically involved in chronic itch. Furthermore, we demonstrate that the ACC→VTA circuit also selectively modulated histaminergic acute itch. Finally, the ACC neurons were shown to predominantly innervate the non-dopaminergic neurons of the VTA. Taken together, our findings uncover a cortex–midbrain circuit for chronic itch-evoked scratching behaviors and shed novel insights on therapeutic intervention.

## Introduction

Itch is defined as an aversive sensation that triggers a desire to scratch [1]. It can be classified into either histaminergic or non-histaminergic itch according to the dependence on histamine receptors [2, 3]. Acute itch serves as an alarm system to warn the body of exogenous danger and prevent potential tissue damage by inducing a scratching response to remove invading irritants. However, when acute itch evolves into a chronic itch, it loses its adaptive function and becomes a debilitating and intractable disease, having a substantial negative impact on a patient’s quality of life due to the unstoppable itch-scratch vicious cycle [2, 4, 5]. A variety of medical conditions are associated with chronic itch, including inflammatory skin disorders [6, 7], chronic kidney disease [8], intrahepatic cholestasis [9, 10], diabetic neuropathy [11, 12], post-herpetic itch [13], and psychogenic itch [14]. Despite the high incidence and huge socioeconomic burden of chronic itch, unfortunately, the optimal treatment against this disease is still lacking [15, 16]. One of the most important reasons for this unmet clinical need is insufficient knowledge of the exact mechanisms underlying the various types of chronic itch. In the past several decades, acute itch-specific molecules, neurons, and neural pathways have gradually been identified [3, 17–20]. However, compared to acute itch, much less attention has been paid to the pathogenic mechanisms of chronic itch, which may hinder advances in clinical therapy.

It is well appreciated that peripherally-derived itch signals are first transmitted to the spinal dorsal horn, which then conveys the pruritic information to higher brain centers for final perception [21, 22]. Previous research efforts have elucidated the etiological mechanisms of chronic itch at peripheral and spinal levels, including the involvement of abnormalities in ion channel expression/activity [23, 24], neuro-immune interaction [25–27], and spinal glial activation [28–30]. However, the central cortical mechanisms of chronic itch remain relatively poorly understood.

The anterior cingulate cortex (ACC) is an important forebrain region involved in many key brain functions, such as learning, memory, sensation, and emotion processing. Multiple forms of synaptic plasticity occurring in the pyramidal neurons of the ACC have been reported to contribute crucially to chronic pain and comorbid anxiety-like symptoms [31–34]. Recent studies have also revealed the circuit basis for the involvement of the ACC in pain and pain-related aversion [35–37]. Intriguingly, relatively less information is now available on the role of ACC in itch, although pain and itch share some similarities in anatomical pathways and protective functions [38, 39]. Notably, the neural projections from the ACC to the dorsal medial striatum have been shown to modulate histaminergic, but not non-histaminergic, acute itch [40]. However, whether the ACC contributes to chronic itch and the potential circuit mechanisms for this contribution remain elusive.

Here, we investigated the functional role of ACC neurons in chronic itch and the underlying circuitry. Combining virus-mediated anterograde/retrograde tracing, c-Fos immunostaining, cell type- or projection-specific chemogenetic manipulations, animal models, and chronic itch-related behavioral assays, we found that ACC neurons significantly modulate chronic itch-evoked scratching behaviors in a cell type-dependent manner. Excitatory neurons in the ACC regulate chronic itch mainly through their downstream projections to the ventral tegmental area (VTA), a midbrain structure recently reported being involved in the central processing of both acute and chronic itch [41, 42]. Our study provides compelling evidence supporting the critical engagement of the ACC→VTA circuit in chronic itch and might shed new insight into intervention strategies for clinical itch therapy.

## Materials and Methods

### Animals

All animal studies and experimental procedures were approved by the Animal Care and Use Committees at Shanghai Tenth People's Hospital and Shanghai Jiao Tong University. Adult (6–12 weeks old) male C57BL/6J mice (Shanghai Laboratory Animal Center, Shanghai), Vgat-Cre (Jackson Laboratory, Bar Harbor, ME), and H2B-GFP mice (Rosa26-loxp-STOP-loxp-H2B-GFP) (gift from Prof. Miao He, Fudan University Shanghai) were used in this study. All mice were group housed (4–5 per cage) under a 12-h light/dark cycle (lights on from 07:00 to 19:00) at a constant temperature and humidity with *ad libitum* food and water. All behavioral experiments were performed during the light phase. The animals were randomly allocated to experimental and control groups.

### Viral Constructs and Stereotaxic Surgery

AAV-hSyn-hM4D(Gi)-mCherry (AAV2/9, 3.18 × 10^12^ genomic copies/mL, AG50475), AAV-CaMKIIα-hM4D(Gi)-mCherry (AAV2/9, 1.56 × 10^13^ genomic copies/mL, H5778), AAV-EF1α-DIO-hM4D(Gi)-mCherry (AAV2/9, 1.38 × 10^13^ genomic copies/mL, HYMBE1370), AAV-Retro-hSyn-mCherry (rAAV, 2.13 × 10^13^ genomic copies/mL, AOV063), AAV-EF1α-DIO-mCherry (AAV2/9, 3.46 × 10^12^ genomic copies/mL, and AG20299) and AAV-Retro-hSyn-Cre (rAAV, 8.18 × 10^12^ genomic copies/mL, CN867) were all made by OBiO Technology, Shanghai, China. AAV1-hSyn-Cre (AAV1, 1.09 × 10^13^ genomic copies/mL, PT0136) was purchased from BrainVTA, Wuhan, China.

Stereotaxic surgery was performed as previously described [42]. Mice were anesthetized with 1% pentobarbital (100 mg/kg, i.p.) and placed on a stereotaxic frame (RWD Life Science, China) with a heating pad to maintain body temperature. A small craniotomy was made using a dental drill through which to inject the virus *via* a glass micropipette (tip diameter: 15 μm–25 μm). Viral injections were targeted into the ACC (AP + 0.62 mm, ML ± 0.26 mm, DV, –1.9 mm) or VTA (AP –3.2 mm, ML ± 0.50 mm, DV, –4.15 mm) according to the Paxinos and Franklin mouse brain atlas (2nd edition). On each side, 300 nL–400 nL of the virus was injected at a rate of 50 nL/min using a hydraulic pump (RWD Apparatus). After the injection, the glass pipette was left in place for 10 min before the withdrawal. The animals were allowed to recover from anesthesia on a heating blanket. Experiments were performed 4–6 weeks after the virus injection. The virus injection sites were verified by *post hoc* immunohistochemistry. Only those animals with correct virus expression were included for further analysis.

### Acute Itch Behavior Test

All animals were handled and acclimatized to the testing room at least three days before any behavioral experiments. The acute itch behavior test was applied as previously described [42–44]. Briefly, mice were shaved on the nape of the neck the day before the test. The animals were placed separately in clear Plexiglas observation boxes on an elevated transparent glass floor and allowed to habituate for 30 min. Pruritogen (histamine: 500 μg/50 μL; chloroquine: 200 μg/50 μL) was then injected intradermally into the back of the neck, and scratching behavior was video recorded for 30 min. The videos were played back on a computer and the total number of scratching bouts was manually counted by trained experimenters who were blind to the treatment groups. A scratch was defined as the lifting of the hind paw toward the injection site and placing it back on the ground or to the mouth.

### Diphenylcyclopropenone (DCP)-Induced Chronic Itch

A contact dermatitis model of chronic itch was developed by applying DCP to the neck skin as described previously [29, 45, 46]. The back of the mouse was shaved and painted with 0.2 mL DCP (1% dissolved in acetone). Seven days after the initial sensitization, 0.2 mL 0.5% DCP was painted on the neck skin daily for two weeks. Scratching behaviors were video recorded for 60 min at the indicated time points or after the final DCP application. The number of scratching episodes was analyzed as described above for the acute itch model.

### Bombesin-saporin Treatment

To ablate the spinal neurons expressing the gastrin-releasing peptide receptor (GRPR), mice were intrathecally injected with bombesin-saporin (400 ng/5 μL) as described previously [44, 45]. Blank-saporin (400 ng/5 μL) was administered in a similar way as a control. Bombesin-saporin or blank-saporin was injected 3 days before DCP modeling, and behavioral or immunohistological experiments were performed 24 days later.

### Open Field Test

Mice were placed in the center of an open box (40 cm^3^ × 40 cm^3^ × 40 cm^3^) and video-taped individually. The animal movement was tracked by an overhead-mounted camera interfaced with a computer running Noldus (Ethovision 3.0) software. Total distance traveled and ambulatory average velocity was measured for 20 min following chemogenetic manipulations.

### Chemogenetic Manipulations

To chemogenetically inactivate all ACC neurons, AAV-hSyn-hM4D(Gi)-mCherry was bilaterally injected into the ACC of C57/BL6J mice. AAV-CaMKIIα-hM4D(Gi)-mCherry was used to selectively inhibit excitatory cingulate neurons. For chemogenetic inhibition of inhibitory GABAergic neurons, AAV-EF1α-DIO-hM4D(Gi)-mCherry was injected into the ACC of Vgat-Cre mice. To selectively inhibit ACC neurons projecting to the VTA, C57/BL6J mice were injected with AAV-Retro-hSyn-Cre in the VTA, and AAV-EF1α-DIO-hM4D(Gi)-mCherry bilaterally in the ACC. To selectively inhibit VTA neurons projecting to the ACC, C57/BL6J mice were injected with AAV-Retro-hSyn-Cre in the ACC, and AAV-EF1α-DIO-hM4D(Gi)-mCherry bilaterally in the VTA. For chemogenetic inhibition of VTA neurons innervated by the ACC, we injected AAV1-hSyn-Cre into the ACC and AAV-EF1α-DIO-hM4D(Gi)-mCherry into the VTA. For all *in vivo* chemogenetic manipulations, mice were injected with clozapine N-oxide (CNO, i.p., 1 mg/kg, Sigma) 30 min prior to acute or chronic itch behavioral tests.

### Slice Electrophysiology

Whole-cell recordings were made from ACC neurons in acute brain slices from mice that had been stereotaxically injected with the required virus. Procedures for slice preparation and electrophysiological recordings followed previous publications [32, 47, 48]. Mice were deeply anesthetized with isoflurane and transcardially perfused with ice-cold n-methyl-d-glucamine (NMDG) artificial cerebrospinal fluid (ACSF) (in mmol/L: 93 NMDG, 93 HCl, 2.5 KCl, 1.25 NaH_2_PO_4_, 10 MgSO_4_, 30 NaHCO_3_, 0.5 CaCl_2_, 25 glucose, 20 HEPES, 5 sodium ascorbate, 3 sodium pyruvate; pH 7.4; osmolarity: 295–305 mOsm). The brain was rapidly dissected, and coronal slices (280 μm) through the ACC were cut on a vibratome (VT1200S, Leica Microsystems). The slices were allowed to recover in oxygenated NMDG ACSF for 10–15 min at 32°C, then transferred to normal oxygenated ACSF (in mmol/L: 126 NaCl, 2.5 KCl, 1.25 NaH_2_PO_4_, 2 MgSO_4_, 10 glucose, 26 NaHCO_3_, 2 CaCl_2_; pH 7.35 when saturated with 95% O_2_/5% CO_2_; osmolarity: 280–300 mOsm) for 1 h at room temperature. Then, the slices were submerged in a recording chamber and continuously perfused with ACSF (2 mL/min–3 mL/min, bubbled with 95% O_2_/5% CO_2_) at 25°C. Cells were visualized under an upright microscope (BX51, Olympus) equipped with differential interference contrast optics and an infrared CCD camera. Patch electrodes (3 MΩ–5 MΩ) were pulled on a micropipette puller (Sutter P-2000) from borosilicate glass (Sutter Instruments) and backfilled with an internal solution containing (in mmol/L): 130 k-gluconate, 8 NaCl, 10 HEPES, 1 EGTA, 2 MgCl_2_, 2 ATP, and 0.2 GTP. Whole-cell current-clamp recordings of ACC neurons identified by their fluorescence were made using a MultiClamp 700B amplifier (Molecular Devices, Sunnyvale, CA, USA). Signals were low-pass-filtered at 2 kHz, digitized at 10 kHz with a Digidata 1440A (Molecular Devices), and recorded using pClamp 10 software. For functional validation of the chemogenetic inhibition, mCherry-labeled neurons were injected with a 250-pA current (500 ms), and the number of activated action potentials was counted at baseline (2 min–5 min) and 10 min after CNO application (5 μmol/L, 2 min). Series and input resistance were monitored throughout the experiments. Data were analyzed offline with Clampfit 10.4 software (Molecular Devices).

### Histology and Fluorescent Immunostaining

The animals were anesthetized with 1% pentobarbital and perfused intracardially with 0.9% saline followed by 4% paraformaldehyde in phosphate-buffered saline (PBS). The brain was then removed and placed in 4% paraformaldehyde buffer at 4°C for overnight fixation, after which the brain was cryoprotected in 30% sucrose solution. Coronal brain sections were cut at 40 μm on a cryostat (VT1000S, Leica). For immunostaining, free-floating sections were first blocked in 0.01 mol/L PBS containing 10% donkey serum and 0.3% Triton X-100 and then incubated with rabbit anti-c-Fos antibody (1:500, Cell Signaling Technology, Cat# 2250) or mouse anti-tyrosine hydroxylase (TH) antibody (1:500, Millipore, Cat# MAB318) at 4°C overnight. After rinsing, the sections were incubated with fluorophore-conjugated secondary antibody (Alexa Fluor 488, donkey anti-rabbit or goat anti-mouse, 1:200; Millipore) for 2 h at room temperature. After three washes, all sections were mounted and cover-slipped with anti-fade reagent with DAPI (ProLong Gold Antifade Reagent with DAPI, ThermoFisher Scientific). All fluorescence images were acquired on a Nikon A1 confocal microscope or an Olympus VS120 microscope using 10× and 20× objectives. Imaging settings remained consistent for all groups.

To detect itch-induced c-Fos expression in the ACC or VTA, we painted the neck skin with DCP 1–1.5 h before perfusion. The number of c-Fos immunoreactive neurons was counted in 3–4 slices from each mouse through different bregma planes of the ACC or VTA. To assess the possible activation of VTA-projecting ACC neurons by DCP, AAV-Retro-hSyn-mCherry was injected into the VTA, then ACC slices were cut after DCP modeling for c-Fos immunostaining. To determine the activation of ACC-innervated VTA neurons by chronic itch, AAV1-hSyn-Cre was injected into the ACC, and AAV-EF1α-DIO-mCherry was infused into the VTA. VTA slices were cut after DCP modeling and stained for c-Fos. To identify the possible innervation into the VTA originating from inhibitory neurons of the ACC, AAV-Retro-hSyn-mCherry was injected into the VTA of Vgat-Cre::H2B-GFP mice and double labeling between mCherry and GFP was analyzed. To evaluate the cell types in the VTA receiving ACC projections, AAV1-hSyn-Cre was injected into the ACC, and AAV-EF1α-DIO-mCherry was infused into the VTA. Then we analyzed the co-labeling between mCherry and immunostained TH to determine the preferential innervation onto the dopaminergic neurons from the ACC. All cell counting was carried out manually with the ImageJ software (NIH Image, version 1.53c) blindly to the experiment group.

### Statistical Analysis

All statistical analyses were performed using Graphpad Prism 9.0. The data were analyzed using either unpaired Student's *t* test, paired Student’s *t* test, or two-way ANOVA followed by the Bonferroni *post hoc* test. All data are presented as the mean ± SEM. *P* <0.05 was considered statistically significant.

## Results

### Cell Type-Dependent Functional Role of ACC Neurons in Chronic Itch

**Fig. 1:**
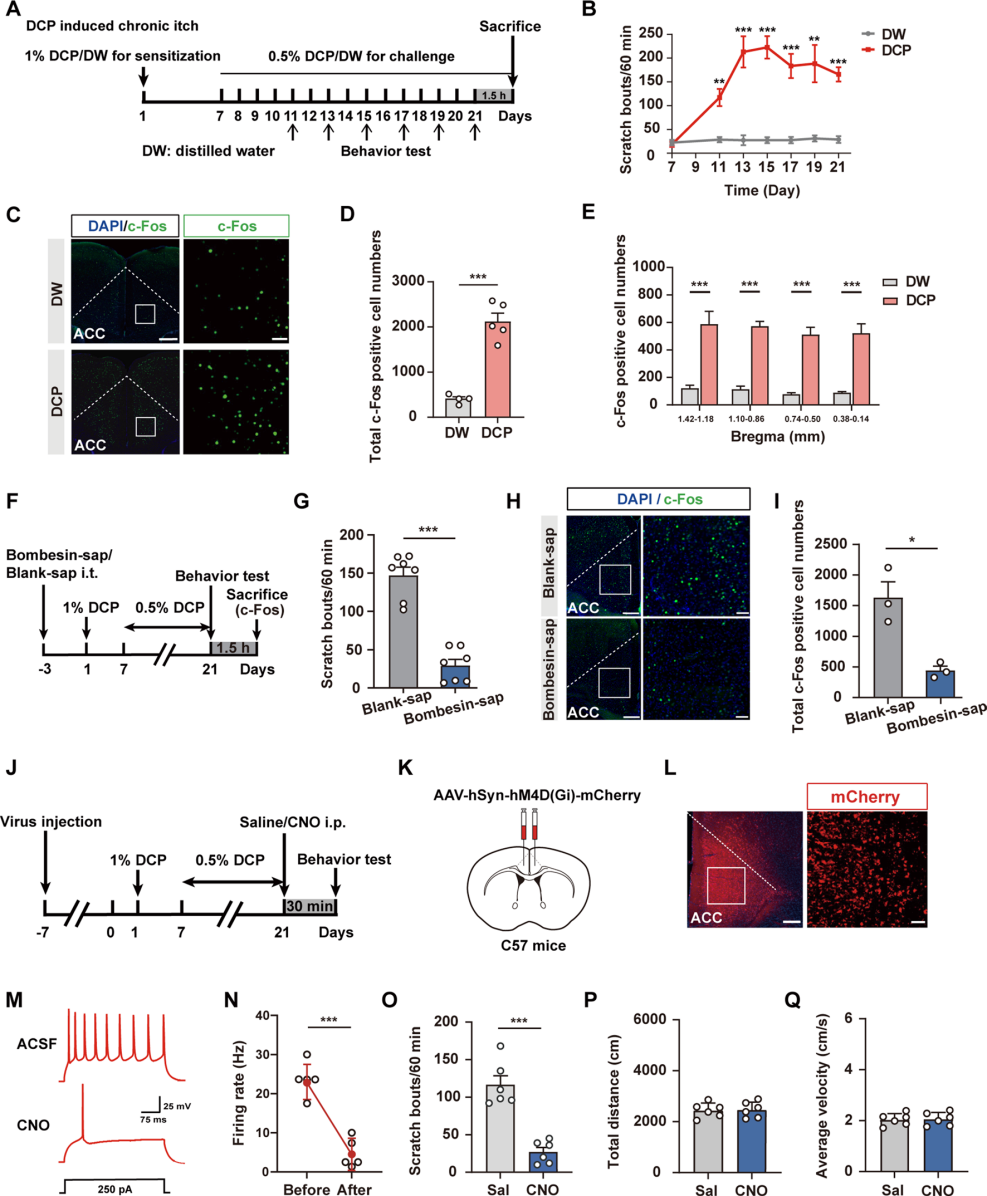
Contribution of ACC neurons to chronic itch-induced scratching behaviors. **A** Schematic showing the experimental timeline for establishing the chronic itch model. DCP, diphenylcyclopropenone; DW, distilled water. **B** Time course of DCP-induced scratching behavior. Numbers of scratching bouts within 60 min were counted. *n* = 5 mice per group; **P <0.01, ***P <0.001; two-way ANOVA followed by Bonferroni *post hoc* analysis. **C** Representative immunostaining images of c-Fos expression in the ACC. Scale bars, 200 μm (left) and 30 μm (right). **D**, **E** Numbers of c-Fos^+^ neurons in total (**D**) and different bregma planes (**E**) of the ACC in the DCP-treated group compared with the DW control group. *n* = 4 or 5 mice; ***P <0.001; unpaired Student’s *t* test for (**D**); two-way ANOVA followed by Bonferroni *post hoc* analysis for (**E**). **F** Schematic showing the experimental timeline for intrathecal ablation of itch-specific GRPR^+^ neurons. Sap, saporin. **G** Scratching behavior evoked by DCP is significantly blocked in mice treated with bombesin-sap compared with blank-sap. ***P <0.001; unpaired Student’s *t* test. **H**, **I** Representative image (**H**) and quantification (**I**) showing a dramatic reduction of DCP-induced c-Fos expression in the ACC when the ascending itch signal is blocked by bombesin-sap. *n* = 3 mice per group; *P <0.05; unpaired Student’s *t* test. Scale bar, 200 μm (left), 50 μm (right). **J** Timeline for chemogenetic inhibition of ACC neuronal activity and behavioral tests. **K** Schematic of the sites of viral injection into the ACC. **L** A representative image showing the expression of hM4Di-mCherry in the ACC. Scale bars, 200 μm (left) and 50 μm (right). **M**, **N** Whole-cell patch clamp recordings reveal that bath application of CNO (5 μmol/L) blocks action potential firing of ACC pyramidal neurons. **M** Representative trace. **N** Quantification data. *n* = 5 neurons. ***P <0.001; paired Student’s *t* test. **O** Quantitative analyses of the number of scratching bouts within 60 min in mice treated with saline or CNO to chemogenetically inactivate ACC neurons. Sal: saline. *n* = 6 mice per group; ***P <0.001; unpaired Student’s *t* test. **P**, **Q** Total distance travelled (**P**) and average velocity (**Q**) in the open field. No significant difference was detected between the two groups. *n* = 6 mice per group. Data are presented as the mean ± SEM.

To explore whether the ACC is involved in chronic itch, we first examined the activation of cingulate neurons using c-Fos as a neuronal activity marker. We generated a mouse model of atopic dermatitis-related chronic itch as previously described [28, 29, 42] and analyzed the number of scratching bouts for 1 h based on video recording (Fig. 1A, B). We observed that repeated painting of DCP onto the neck skin caused a gradual increase in the scratching behavior that peaked at 15 days and lasted until 21 days after the initial DCP sensitization (Fig. 1B). Meanwhile, immunofluorescence staining showed that the number of c-Fos^+^ neurons increased significantly in different bregma planes of the ACC in animals subjected to DCP-induced chronic itch compared with the distilled water (DW)-treated control animals (Fig. 1C–E). Here, mechanical stimulation resulting from DCP-induced scratching motion may cause c-Fos activation. DCP-induced local inflammation may also introduce nonspecific effects on c-Fos staining. To ascertain that the c-Fos labelling in the ACC was due to “itch” but no other confounding factors, we performed experiments involving the ablation of spinal itch-specific GRPR^+^ neurons by intrathecal injection of bombesin-saporin [44, 45]. As expected, this manipulation significantly reduced the DCP-induced scratching behavior compared to the blank-saporin (Fig. 1F, G), demonstrating the successful inhibition of ascending spinal GRPR-dependent itch pathway. Importantly, intrathecal injection of bombesin-saporin dramatically reduced c-Fos expression in the ACC (Fig. 1H, I), suggesting that the enhanced c-Fos labeling induced by DCP is indeed due to the presence of “itch”; this is also consistent with our previous work revealing the enhanced excitatory synaptic transmission of the ACC after chronic itch [48].

To determine the overall contribution of the ACC to chronic itch processing, we applied the chemogenetic methods of designer receptors exclusively activated by designer drugs (DREADDs) to manipulate the ACC neuronal activity. Adeno-Associated Virus (AAV) vectors carrying neuron-specific inhibitory DREADDs fused with mCherry (AAV-hSyn-hM4Di-mCherry) were microinjected bilaterally into the ACC 7 days prior to DCP modeling (Fig. 1J, K). We confirmed the correct location of virus injection by *post hoc* observation of the mCherry fluorescence in the ACC (Fig. 1L). The efficiency of silencing ACC neurons with hM4Di was validated by recording neuronal activity in brain slices. We found that bath application of Clozapine-N-oxide (CNO) (5 μmol/L) suppressed the spiking activity of hM4Di^+^ neurons (Fig. 1M, N). We tested scratching behaviors 30 min after the intraperitoneal injection of CNO (1 mg/kg) or saline at 21 days after DCP treatment. Chemogenetic inactivation of ACC neurons significantly suppressed DCP-evoked scratching behaviors (Fig. 1O), as shown by the reduced number of scratching bouts (27.00 ± 6.09) compared to the saline control (116.50 ± 12.11). The locomotor function of these animals remained unaltered (Fig. 1P, Q). These results suggest that ACC neurons are critically involved in chronic itch-evoked repetitive scratching behaviors.

**Fig. 2:**
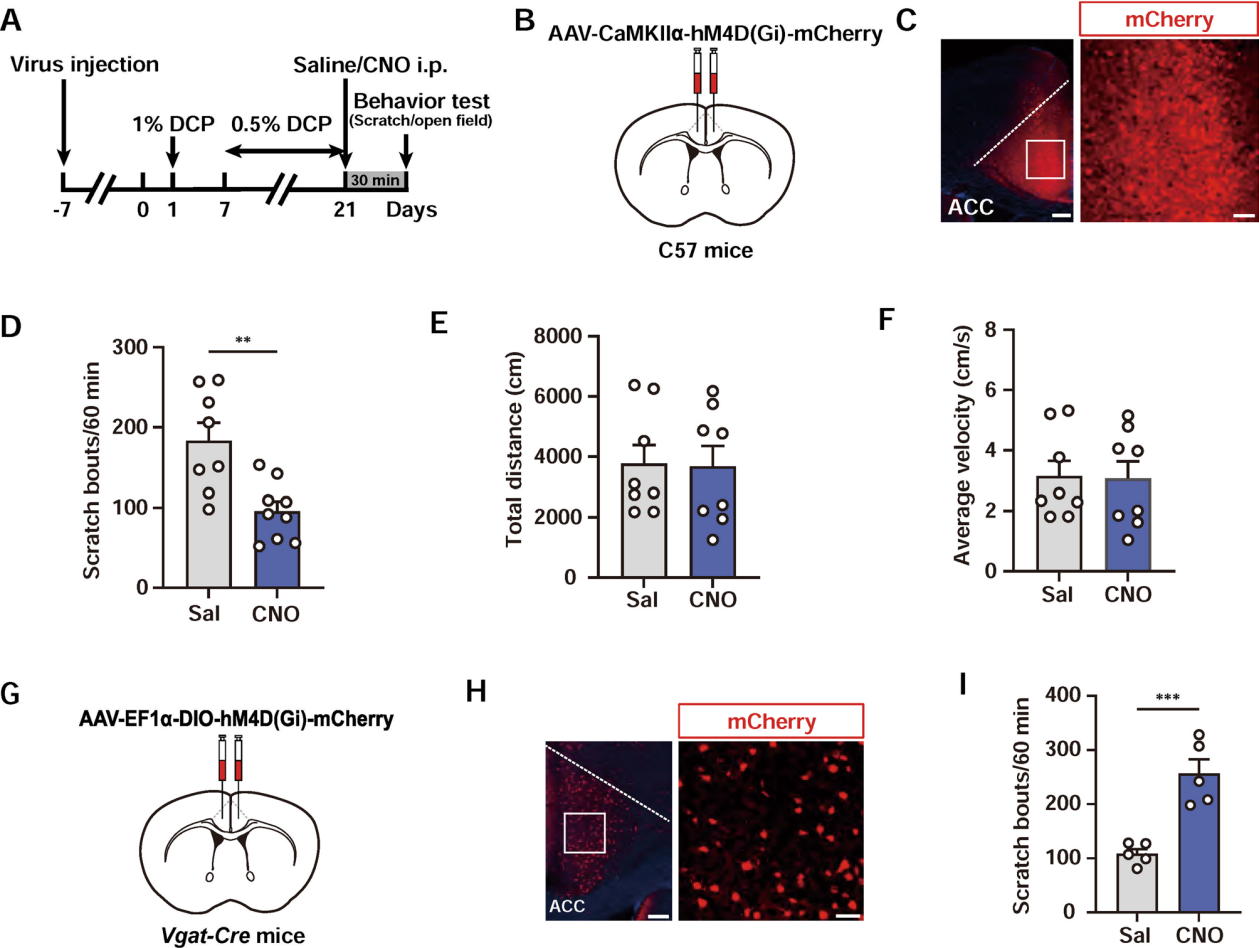
ACC modulates chronic itch in a cell type-dependent manner. **A** Schematic showing the experimental timeline for selective chemogenetic inhibition of excitatory neurons in the ACC. **B** Scheme for specific infection of excitatory neurons in the ACC with hM4Di. **C** A representative image illustrating the expression of hM4Di-mCherry in the ACC. Scale bars, 200 μm (left) and 50 μm (right). **D** Quantitative analyses of the number of scratching bouts within 60 min in mice treated with saline or CNO to chemogenetically inactivate excitatory ACC neurons. *n* = 8 or 9 mice; **P <0.01; unpaired Student’s *t* test. **E**, **F** Total distance travelled (**E**) and average velocity (**F**) in the open field. No significant difference was found between the two groups. *n* = 8 mice per group. **G** Scheme for specific infection of inhibitory neurons in the ACC with hM4Di. **H** Histological verification of viral infection. Scale bars, 200 μm (left) and 50 μm (right). **I** Chemogenetic suppression of inhibitory neurons in the ACC enhances scratching behaviors in chronic itch. *n* = 5 mice; ***P <0.001; unpaired Student’s* t* test. Data are presented as the mean ± SEM.

Different cell types in the ACC have been shown to play distinct and even opposite roles in sensory processing [49]. To assess the cell type-specific roles of cingulate neurons in chronic itch, we stereotaxically infused AAV-CaMKIIɑ-hM4Di-mCherry to selectively inhibit glutamatergic neurons in the ACC (Fig. 2A–C). We found that chemogenetic inhibition of ACC excitatory neurons significantly reduced scratching behaviors in chronic itch (Fig. 2D), with the number of scratching bouts decreasing to 95.56 ± 12.07 compared with the saline control (183.40 ± 22.30). Inhibition of ACC excitatory neurons did not affect the behavioral performance in the open field test, indicating the lack of a clear effect on locomotor activity (Fig. 2E, F). To test the role of GABAergic neurons in the ACC during chronic itch, we injected a Cre-dependent AAV (AAV-EF1ɑ-DIO-hM4Di-mCherry) bilaterally into the ACC of Vgat-Cre mice (Fig. 2G, H). Selective inhibition of ACC inhibitory neurons resulted in an evident increase in DCP-evoked scratching behaviors (CNO: 256.60 ± 26.12; Saline: 108.40 ± 8.86; Fig. 2I). These results suggest that excitatory and inhibitory neurons in the ACC have different influences on chronic itch-related scratching behaviors.

### Chronic Itch Activates the ACC→VTA Projection

**Fig. 3:**
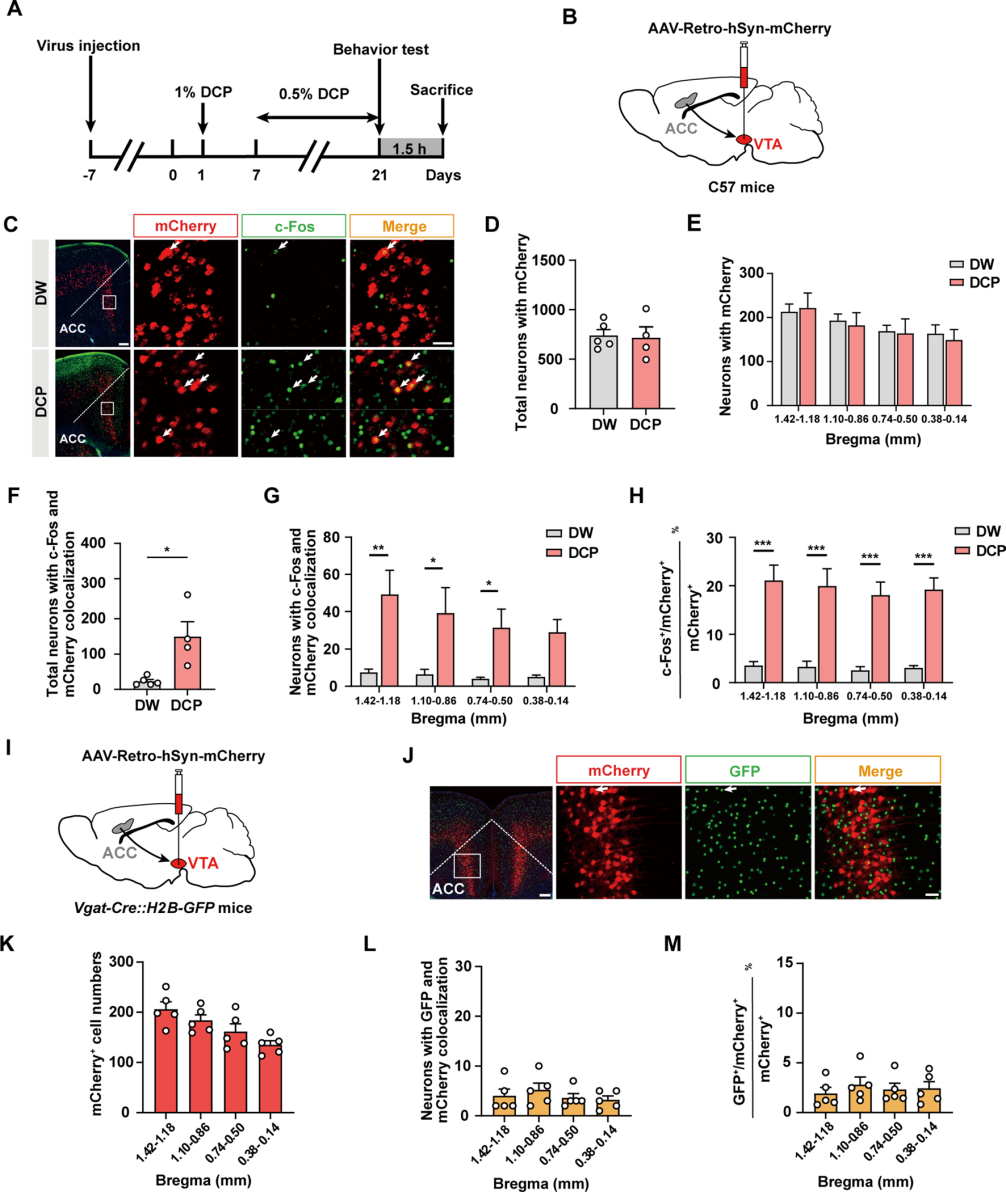
Activation of the ACC→VTA excitatory projection by chronic itch. **A** Schematic showing the timeline of the immunostaining experiments. **B** Scheme for retrograde labelling of VTA-projecting ACC neurons. **C** Representative images of c-Fos co-staining with mCherry in the ACC for both DW- and DCP-treated groups. Arrows indicate c-Fos^+^/mCherry^+^ neurons. Scale bars, 200 μm (left) and 50 μm (right). **D**, **E** Numbers of mCherry^+^ neurons in total (**D**) and different sections (**E**) of the ACC. *n* = 5 or 4 mice per group. **F**, **G** Numbers of c-Fos^+^/mCherry^+^ neurons in total (**F**) and different sections (**G**) of the ACC. *n* = 5 or 4 mice; *P <0.05, **P <0.01; unpaired Student’s *t* test for (**F**); two-way ANOVA followed by Bonferroni *post hoc* analysis for (**G**). **H** Percentage of c-Fos^+^/ mCherry^+^ neurons in mCherry^+^ neurons for different bregma planes of the ACC. *n* = 5 or 4 mice; ***P <0.001; two-way ANOVA followed by Bonferroni *post hoc* analysis. **I** Scheme for retrograde labelling of VTA-projecting ACC neurons in Vgat-Cre::H2B-GFP mice. **J** Representative images of GFP co-staining with mCherry in the ACC. Arrows indicate GFP^+^/mCherry^+^ neurons. Scale bars, 200 μm (left) and 50 μm (right). **K** Summary of the numbers of mCherry^+^ neurons in different sections of the ACC. *n* = 5 mice per group. **L** Numbers of GFP^+^/mCherry^+^ neurons in different parts of the ACC. *n* = 5 mice per group. **M** Percentage of GFP^+^/mCherry^+^ neurons in mCherry^+^ neurons for different sections of the ACC. Few co-labeled neurons are detected. *n* = 5 mice per group. Data are presented as mean ± SEM.

Previous studies have demonstrated a reciprocal inter-connection between ACC and VTA [50, 51]. Human imaging studies indicate the contribution of both areas to itch processing [52–55]. Therefore, we next asked if the ACC**→**VTA projection could be activated by chronic pruritogenic stimuli. To this end, we injected AAV-Retro-hSyn-mCherry into the VTA and tested c-Fos expression co-staining with mCherry 4 weeks later (Fig. 3A–C). We found that the total number of mCherry^+^ neurons in the DCP group did not differ from the control group (Fig. 3D, E). However, there were more c-Fos^+^/mCherry^+^ neurons in the DCP-treated animals (Fig. 3F, G). Moreover, DCP elicited an increased percentage of c-Fos^+^/mCherry^+^ neurons across all bregma planes of the ACC (Fig. 3H). These data indicate a dramatic activation of the ACC neurons projecting to the VTA under the conditions of chronic itch.

Given the differential modulation of DCP-induced scratching behaviors by excitatory and inhibitory neurons in the ACC (Fig. 2), we then examined the cell type of the ACC neurons projecting to the VTA. We injected AAV-Retro-hSyn-mCherry into the VTA of Vgat-Cre::H2B-GFP mice and counted the number of GFP^+^/mCherry^+^ neurons (Fig. 3I, J). No significant difference was detected in the number of mCherry^+^ neurons between different sections of the ACC (Fig. 3K), suggesting no preferential innervation of the VTA along the rostral-causal axis. Importantly, few GFP^+^ neurons, denoting GABAergic neurons, were found to co-localize with mCherry^+^ neurons, marking the cortical neurons projecting to the VTA (Fig. 3L, M). Thus, most of the ACC neurons innervating the VTA are excitatory projection neurons.

**Fig. 4:**
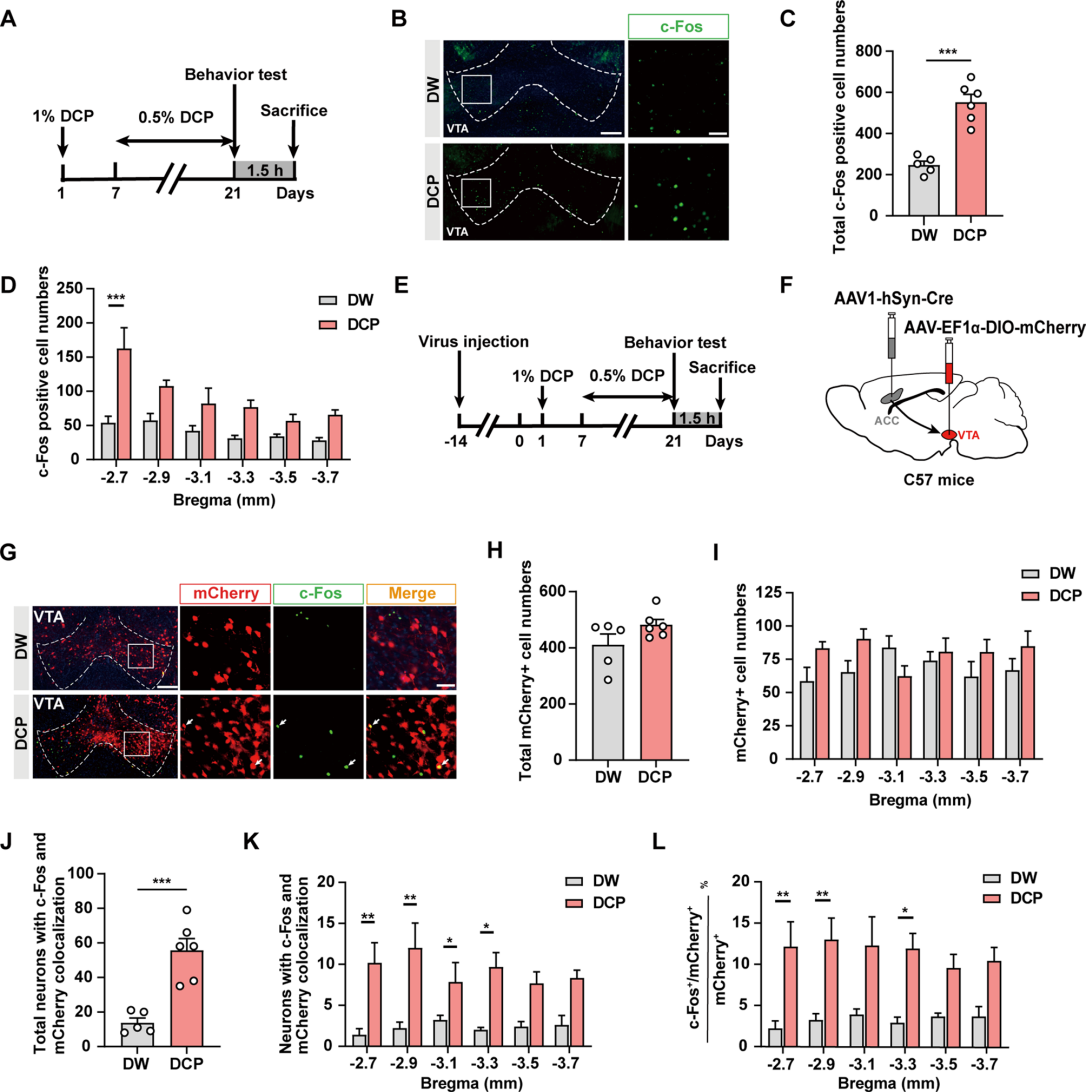
VTA neurons are significantly activated during chronic itch. **A** Timeline of the c-Fos immunostaining experiments. **B** Representative images of c-Fos expression in the VTA for both DW- and DCP-treated groups. Scale bars, 200 μm (left) and 50 μm (right). **C**, **D** Numbers of c-Fos^+^ neurons in total (**C**) and different sections (**D**) of the VTA. *n* = 5 or 6 mice; ***P <0.001; unpaired Student’s *t* test for (**C**); two-way ANOVA followed by Bonferroni *post hoc* analysis for (**D**). **E** Schematic showing the timeline of the immunostaining experiments. **F** Scheme for specific labeling of ACC-innervated VTA neurons. **G** Representative images of c-Fos co-staining with mCherry in the VTA. Arrows indicate c-Fos^+^/mCherry^+^ neurons. Scale bars, 200 μm (left) and 50 μm (right). **H**, **I** Numbers of mCherry^+^ neurons in total (**H**) and different parts (**I**) of the VTA. *n* = 5 or 6 mice. **J**, **K** Numbers of c-Fos^+^/ mCherry^+^ neurons in total (**J**) and different sections (**K**) of the VTA. *n* = 5 or 6 mice; *P <0.05, **P <0.01, ***P <0.001; unpaired Student’s *t* test for (**J**); two-way ANOVA followed by Bonferroni *post hoc* analysis for (**K**). **L** Percentage of c-Fos^+^/mCherry^+^ neurons in mCherry^+^ neurons for different parts of the VTA. *n* = 5 or 6 mice; *P <0.05, **P <0.01; two-way ANOVA followed by Bonferroni *post hoc* analysis. Data are presented as the mean ± SEM.

Since the VTA-projecting ACC neurons are strongly activated by DCP-induced chronic itch, and these long-range projection neurons are mostly excitatory (Fig. 3), it is naturally assumed that the downstream VTA neurons would also be activated. Indeed, the expression of c-Fos in different bregma planes of the VTA was significantly higher in the DCP group than in the DW group (Fig. 4A–D). Moreover, we injected AAV1-hSyn-Cre into the ACC and AAV-EF1ɑ-DIO-mCherry into the VTA to label VTA neurons that received projections from the ACC (Fig. 4E, F). Although the number of mCherry^+^ neurons was not altered by DCP-evoked chronic itch, the number and percentage of c-Fos^+^/mCherry^+^ neurons were significantly higher within different rostro-caudal segments of the VTA in the DCP group than in the DW control (Fig. 4G–L). Taken together, these results suggest a clear activation of the ACC→VTA circuit under chronic itch conditions.

### The ACC→VTA Projection Regulates Scratching Behaviors in Chronic Itch

**Fig. 5:**
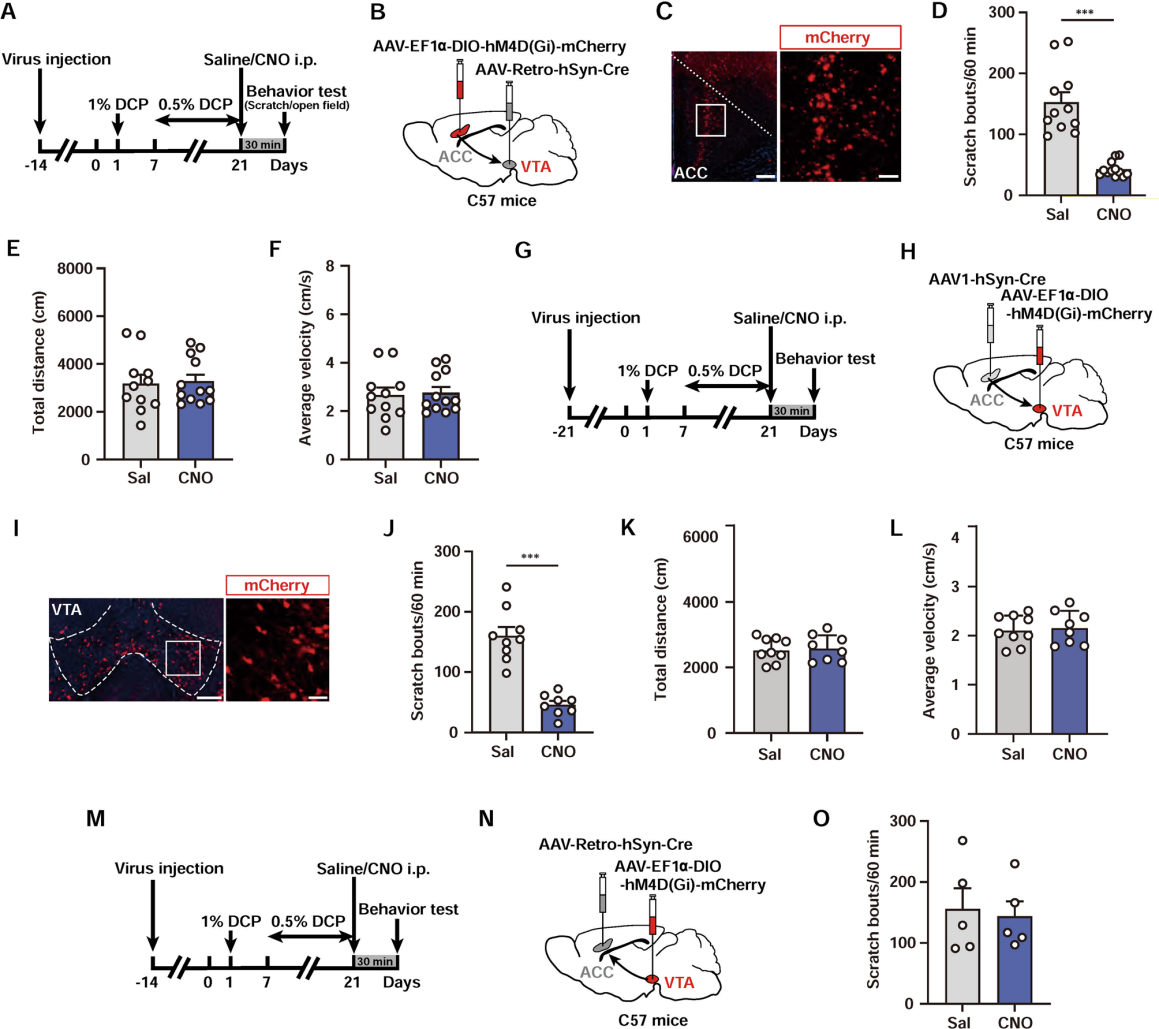
The ACC→VTA projection is required for chronic itch-induced scratching. **A** Experimental timeline for chemogenetic suppression of VTA-projecting ACC neurons in chronic itch. **B** Scheme for specific infection of VTA-projecting ACC neurons with hM4Di. **C** Histological verification of the viral infection. Scale bars, 200 μm (left) and 30 μm (right). **D** Chemogenetic inhibition of ACC neurons that project to VTA results in a significant decrease in DCP-evoked scratching behaviors. *n* = 11 or 12 mice; ***P <0.001; unpaired Student’s *t* test. **E**, **F** Total distance travelled (**E**) and average velocity (**F**) in the open field. No significant difference was detected between the two groups. *n* = 11 or 12 mice. **G** Experimental timeline for chemogenetic suppression of ACC-innervated VTA neurons in chronic itch. **H** Scheme for specific infection of ACC-recipient VTA neurons with hM4Di. **I** Histological verification of the viral infection. Scale bars, 200 μm (left) and 50 μm (right). **J** Chemogenetic inhibition of VTA neurons that receive inputs from the ACC strongly attenuates scratching behaviors in chronic itch. *n* = 9 or 8 mice; ***P <0.001; unpaired Student’s *t* test. **K**, **L** Total distance travelled (**K**) and average velocity (**L**) in the open field. No significant difference was detected between the two groups. *n* = 9 or 8 mice. **M** Experimental timeline for chemogenetic inhibition of the VTA→ACC projection. **N** Scheme for specific infection of ACC-projecting VTA neurons with hM4Di. **O** Chemogenetic inactivation of the VTA→ACC projection has no effect on DCP-evoked scratching behaviors. *n* = 5 mice per group; unpaired Student’s *t* test. Data are shown as the mean ± SEM.

Based on previous results demonstrating the activation of the ACC→VTA circuit by chronic itch, we next investigated whether manipulation of this projection can affect the behavioral outputs. We used two viral strategies to selectively inhibit this circuit by chemogenetic approaches. First, we delivered AAV-Retro-hSyn-Cre [56] into the VTA and bilaterally infected VTA-projecting ACC neurons with a Cre-dependent virus encoding the neuronal inhibitor DREADD (AAV-EF1ɑ-DIO-hM4Di-mCherry) (Fig. 5A–C). ACC neurons projecting to the VTA were chemogenetically inhibited *via* i.p. injection of CNO 30 min prior to the behavioral test at 21 days after DCP modelling (Fig. 5A). Inhibition of VTA-projecting ACC neurons attenuated DCP-evoked scratching behaviors (CNO: 42.83 ± 3.60; Saline: 152.60 ± 16.43; Fig. 5D). By contrast, the locomotor activity of these mice was not significantly influenced (Fig. 5E, F).

In addition to the VTA, the ACC neurons that project to the VTA may send collaterals to other itch-related brain regions [17, 40]. Thus, the antipruritic effects of chemogenetic inhibition of VTA-projecting ACC neurons might be due to altered functions in other downstream areas rather than in the VTA. To further confirm the necessity of the ACC→VTA pathway, we next injected the monosynaptic anterograde transport virus AAV1-hSyn-Cre [57] into the ACC and infected the VTA postsynaptic neurons with AAV-EF1ɑ-DIO-hM4Di-mCherry (Fig. 5G–I). We found that specific inhibition of ACC-recipient postsynaptic neurons in the VTA also significantly decreased DCP-induced scratching behaviors (CNO: 45.75 ± 6.32; Saline: 160.0 ± 14.52; Fig. 5J) without affecting locomotion (Fig. 5K, L). Interestingly, chemogenetic suppression of the ACC-projecting VTA neurons did not affect the numbers of DCP-induced scratching episodes (Fig. 5M–O), indicating the selectivity of the unidirectional ACC→VTA projection, but not the opposite direction, in modulating chronic itch.

### The ACC→VTA Projection Modulates Histaminergic Itch

**Fig. 6:**
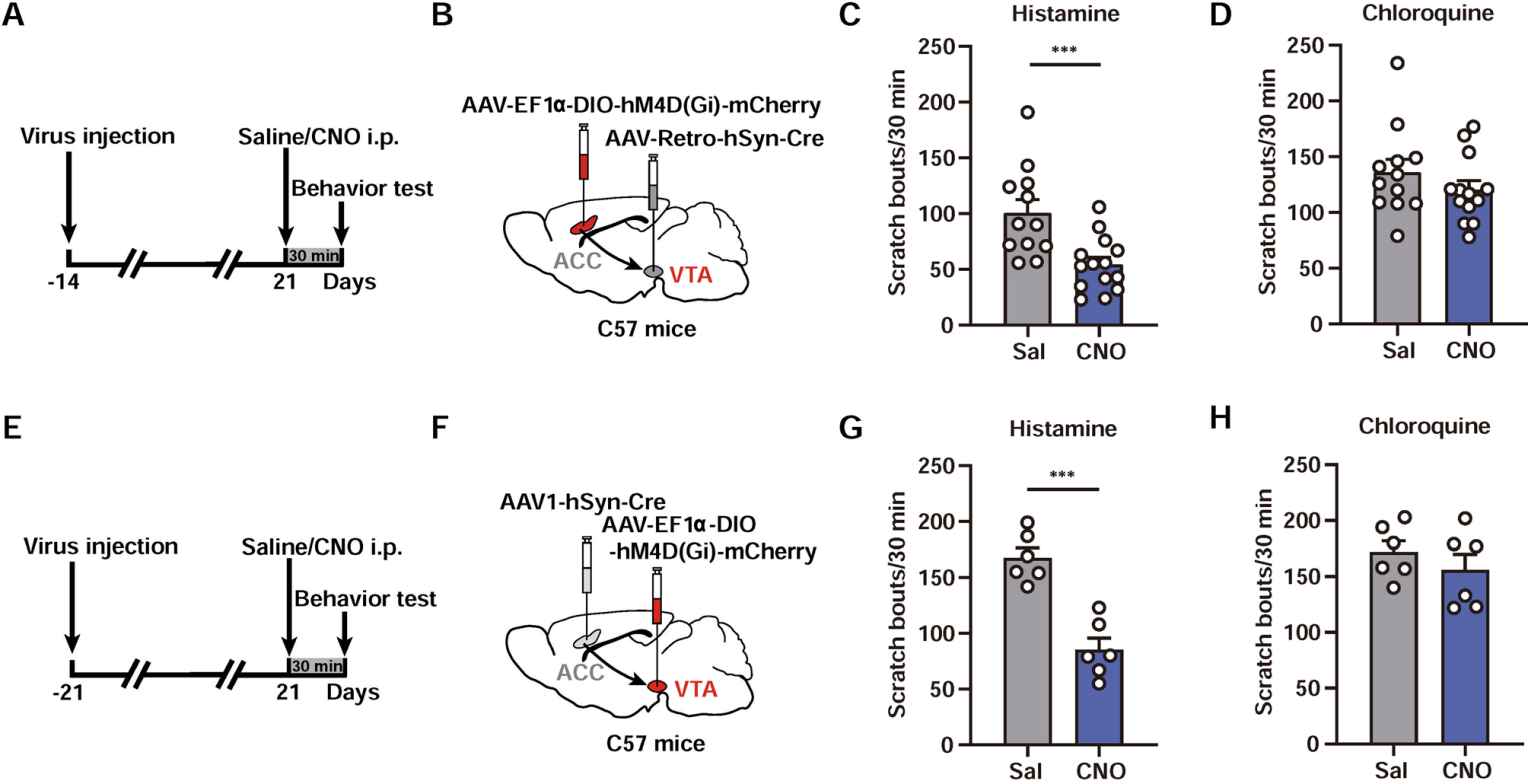
The ACC→VTA circuit modulates histaminergic itch sensation. **A** Experimental timeline for chemogenetic suppression of VTA-projecting ACC neurons in acute itch. **B** Scheme for specific infection of VTA-projecting ACC neurons with hM4Di. **C**, **D** Chemogenetic inhibition of ACC neurons that project to the VTA causes a significant decrease in histamine- (**C**) but not chloroquine-evoked (**D**) scratching behaviors. *n* = 12–14 mice per group; ***P <0.001; unpaired Student’s *t* test. **E** Experimental timeline for the chemogenetic suppression of ACC-innervated VTA neurons in acute itch. **F** Scheme for specific infection of ACC-recipient VTA neurons with hM4Di. **G**, **H** Chemogenetic inhibition of VTA neurons that receive inputs from the ACC strongly attenuates histamine- (**G**) but not chloroquine-induced (**H**) scratching behaviors. *n* = 6 mice per group; ***P <0.001; unpaired Student’s *t* test. Data are shown as the mean ± SEM.

The results presented above show the activation and involvement of the ACC→VTA projection in DCP-evoked chronic itch. Both ACC and VTA neurons have been reported to contribute to acute itch as well [41, 42, 58]. Therefore, we next asked whether the ACC→VTA pathway also modulates acute itch. We applied a similar virus-mediated chemogenetic strategy to selectively inhibit the VTA-projecting neurons in the ACC, by injecting AAV-Retro-hSyn-Cre into the VTA and Cre-dependent AAV-EF1ɑ-DIO-hM4Di-mCherry into the ACC (Fig. 6A, B). Five weeks after viral injection, CNO was administered i.p. 30 min before behavioral tests of histamine- and chloroquine-induced acute itch (Fig. 6A). The results clearly showed a dramatic reduction of histaminergic itch by the ACC→VTA inhibition (CNO: 54.50 ± 6.45; Saline: 100.80 ± 11.67; Fig. 6C). However, the chloroquine-evoked, histamine-independent itch was not significantly altered (CNO: 120.2 ± 8.32; Saline: 136.1 ± 11.56; Fig. 6D). To further confirm the effect, we delivered AAV1-hSyn-Cre into the ACC and infused AAV-EF1ɑ-DIO-hM4Di-mCherry into the VTA (Fig. 6E, F). Chemogenetic inactivation of the VTA neurons innervated by the ACC yielded a similar suppression of histamine- but not chloroquine-induced scratching behaviors (Fig. 6G, H). These results suggest that the ACC→VTA projection plays a positive regulatory role in histaminergic acute itch.

### ACC Projections Predominantly Target Non-dopaminergic Neurons in the VTA

**Fig. 7:**
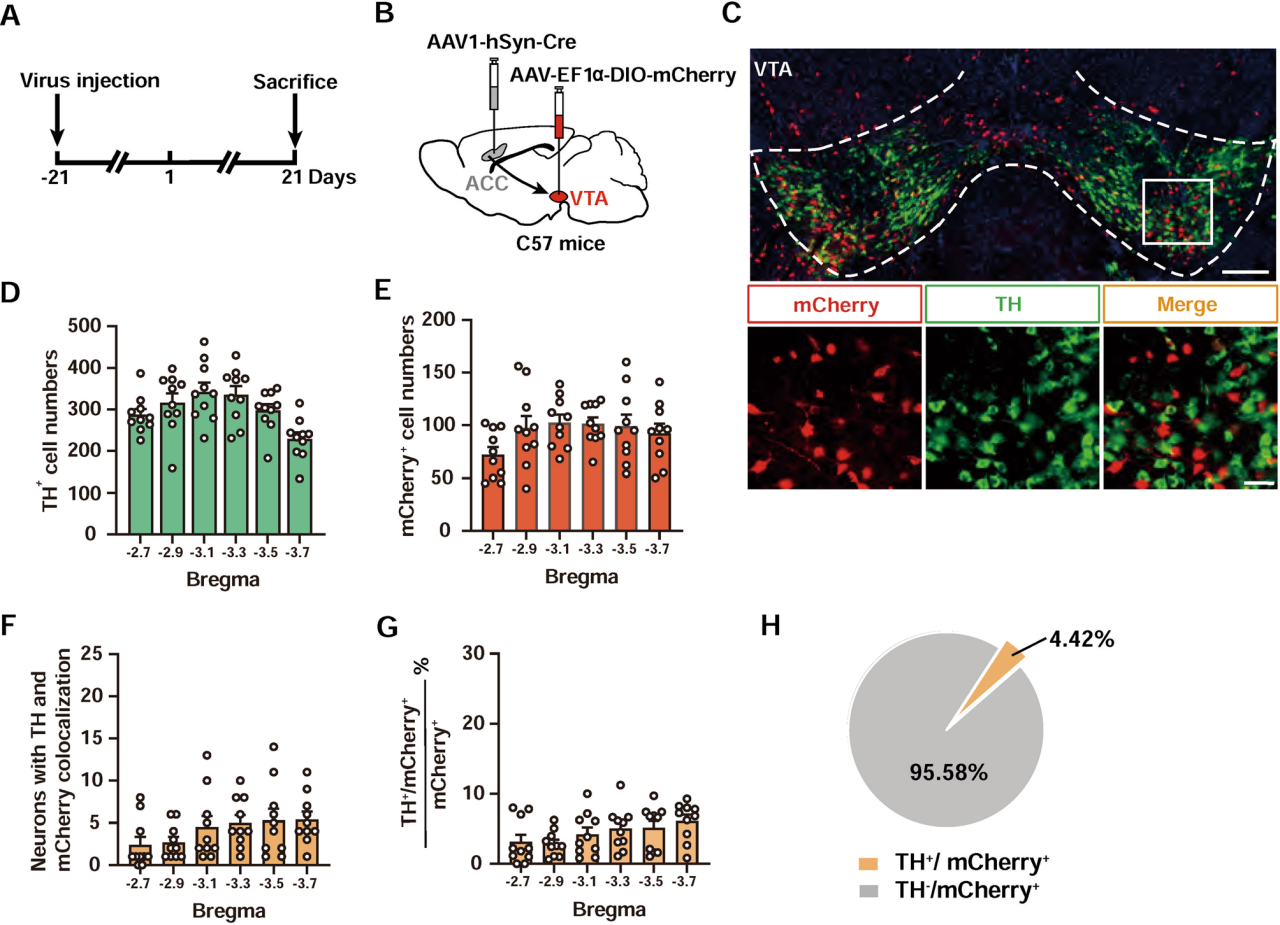
ACC predominantly projects to non-dopaminergic neurons in the VTA. **A** Schematic showing the timeline for virus injection and TH co-staining experiments. **B** Scheme for specific labeling of ACC-innervated VTA neurons with mCherry. **C** Representative images of mCherry co-staining with TH in the VTA. Scale bars, 200 μm (upper) and 50 μm (lower). **D**, **E** Numbers of TH^+^ (**D**) and mCherry^+^ (**E**) neurons in different sections of the VTA. *n* = 10 mice. **F**, **G** Numbers (**F**) and percentage (**G**) of TH^+^/mCherry^+^ neurons in different parts of the VTA. *n* = 10 mice. **H** Pie chart showing minimal expression of mCherry in TH^+^ neurons in the VTA.

Our recent work reported distinct contributions of two major neuronal types in the VTA, namely GABA and dopamine (DA) neurons, in encoding aversive and hedonic aspects of the itch-scratch cycle, respectively [42]. To investigate which cell type of the VTA the ACC pyramidal neurons mainly innervate, we injected anterograde AAV1-hSyn-Cre into the ACC and AAV-EF1ɑ-DIO-mCherry into the VTA, and then performed immunofluorescence staining of tyrosine hydroxylase (TH) 6 weeks later (Fig. 7A, B). Interestingly, we observed a few cells co-stained with mCherry and TH (Fig. 7C–G). Only 4.42% of mCherry^+^ neurons were also TH^+^ (Fig. 7H), even though DA neurons are the most abundant cells in the VTA [59]. Thus, it is conceivable that ACC predominantly projects to non-DA neurons of the VTA to control the chronic itch.

## Discussion

Itch is an unpleasant sensation that elicits the desire to scratch [1, 18, 19]. Although acute itch is an important alarm system to protect the body from harmful external irritants by triggering the scratching response, pathological chronic itch is a serious and intractable disease leading to excessive and repetitive scratching and causing much suffering to the patient [4, 16]. However, the precise higher brain mechanisms underlying the pathophysiology of chronic itch remain poorly understood. Here, we demonstrated the fundamental role of ACC neurons in modulating scratching behaviors in a mouse model of atopic dermatitis. Repeated painting of DCP on the neck skin led to a marked increase in the numbers of c-Fos-immunopositive neurons in the ACC across the different bregma planes. Selective inhibition of excitatory cingulate neurons by the chemogenetic approach caused a remarkable reduction in DCP-evoked scratching bouts, whereas similar inhibition of the GABAergic neurons in the ACC significantly increased scratching, indicating that ACC neurons regulate chronic itch in a cell type-specific manner. It is of great interest to note that a similar cell type-dependent role of ACC neurons in chronic pain has also been illustrated using optogenetic approaches to selectively modulate excitatory pyramidal neurons and inhibitory interneurons in the ACC [49]. These findings add to the accumulating evidence indicating a general cell type-dependent function of ACC neurons in somatosensation and sensory disorders. In addition, the current demonstration of the involvement of cingulate neurons in DCP-induced scratching behaviors is also consistent with our previous report showing a potentiation of excitatory synaptic transmission in the ACC by chronic itch [48].

Since the currently-available pharmacological agents do not work well for treating chronic itch [16], we hypothesize that a unique circuit mechanism might be involved in its pathogenesis. Studying the neural circuit of a brain disease raises the possibility of revealing some converging points of inter-neuron connections for targeting through various neuromodulation methods such as deep-brain stimulation [60] or transcranial magnetic stimulation [61]. These non-drug approaches may work together with the classical drugs to achieve a better therapeutic outcome. In this study, we report the important role of the ACC→VTA projection for chronic itch-induced excessive scratching behaviors. This conclusion is based on three independent lines of evidence. First, virus-mediated anterograde or retrograde tracing and c-Fos immunohistochemical staining showed that both ACC neurons projecting to the VTA and the VTA neurons innervated by ACC were significantly activated under the condition of DCP-evoked chronic itch. Second, projection-specific chemogenetic inhibition of the ACC neurons projecting to the VTA, mainly excitatory pyramidal neurons, effectively suppressed DCP-induced scratching. Not surprisingly, selective inhibition of the VTA neurons innervated by the ACC resulted in a similar reduction of the scratching behavior. Finally, no appreciable changes were detected if we selectively inhibited the VTA neurons projecting to the ACC. Collectively, these data jointly support the hypothesis that ACC neurons control chronic itch, at least in part, through their downstream projections to the VTA, although putative roles of other ACC-centered circuits in itch chronicity cannot be ruled out.

The above results are reminiscent of our recent publication illustrating the differential roles of VTA GABA and DA neurons in encoding distinct emotional components of itch [42]. In that study, using fiber photometry, optogenetics, and newly-established behavioral paradigms, we demonstrated that VTA GABA neurons signal aversion to itch to trigger the scratching response, whereas VTA DA neurons encode scratching-associated reward to sustain the recurrent scratching episodes. Importantly, the dissociable role of VTA GABA and DA neurons also applies to the mouse model of chronic itch [42]. Here, we found that ACC pyramidal neurons predominantly innervate the non-DA neurons of the VTA, implying that the ACC→VTA circuit may largely encode the aversive component of the itch experience to drive repetitive scratching behaviors. Future studies, however, are certainly needed to test this hypothesis. It is also of primary importance to elucidate the possible synaptic plasticity changes within the ACC→VTA circuit under chronic itch conditions.

One notable finding of the present study is that the ACC→VTA circuit equally modulates histamine-evoked acute itch. Chemogenetic inhibition of the projection from the ACC to the VTA led to a significant decrease in histamine- but not chloroquine-induced scratching behaviors. These results are in agreement with previous functional brain imaging studies showing activation of the ACC during pruritogenic stimulation [52, 62, 63]. Consistent with this, circuit interrogations in mice revealed that the neural projections from the anteromedial thalamic nucleus to the ACC and then to the dorsal medial striatum constituted a critical circuit element for the modulation of histaminergic itch [40, 58]. Furthermore, genetic or pharmacological blockade of GluK1-containing kainate receptors in the ACC substantially reduces pruritogen-induced scratching behaviors [64]. Thus, the present results, together with previous findings, provide a rather complete scenario of the circuit/molecular basis underpinning the modulation of itch by ACC neurons, although possible involvement of other ACC-centered circuits or ACC-enriched molecules cannot be excluded. Interestingly, the current manipulation of the ACC→VTA projection failed to affect chloroquine-induced, histamine-independent itch. The exact reasons for the apparent discrepancy are not clear but may be ascribed to differences in the neural pathways for the transmission of histaminergic and non-histaminergic itch signals [65–67]. Consistent with this, manipulations of the thalamic→ACC or the ACC→striatum projections similarly do not have any significant effect on chloroquine-induced acute itch [40, 58]. Thus, these negative data suggest the potential contribution of other key brain regions or other ACC-innervated downstream structures to the regulation of histamine-independent itch.

In conclusion, we report the cell type-dependent functions of ACC neurons in modulating chronic itch by sending projections to the VTA. These results not only extend our understanding of the essential role of ACC neurons and circuits in chronic itch but also provide potential novel therapeutic strategies that involve the neuromodulation of aberrant cortical circuits to treat diseases associated with chronic itch.
